# Obesity, Depression, and Antidepressant Use: Analyzing Metabolic Side Effects in US Adults Using the National Health and Nutrition Examination Survey (NHANES)

**DOI:** 10.7759/cureus.88143

**Published:** 2025-07-17

**Authors:** Amaka S Odega, Damilola A Adeyemo, Joseph E Igetei, Tochukwu W Okahia, Victoria Aliu, Okelue E Okobi

**Affiliations:** 1 Psychiatry, Sault Area Hospital, Sault Ste. Marie, CAN; 2 Psychiatry, Northern Ontario School of Medicine (NOSM) University, Thunder Bay, CAN; 3 Research, Texas A&M University, Corpus Christi, USA; 4 General Medicine, International University of the Health Sciences, Basseterre, KNA; 5 Psychiatry, Leeds and York Partnership NHS Foundation Trust, Leeds, GBR; 6 Psychiatry, Ambrose Alli University/Irrua Specialist Teaching Hospital, Ekpoma, NGA; 7 Family Medicine, IMG Research Academy and Consulting, Homestead, USA; 8 Family Medicine, Larkin Community Hospital Palm Springs Campus, Hialeah, USA; 9 Family Medicine, Lakeside Medical Center, Belle Glade, USA

**Keywords:** antidepressant use, body mass index (bmi), depression, metabolic outcomes, nhanes, obesity

## Abstract

Background: Antidepressant use is increasingly prevalent, raising concerns about its potential impact on metabolic health, particularly in individuals with depression. This study investigated the association between antidepressant use and metabolic outcomes in a nationally representative US adult population.

Methods: Data from the National Health and Nutrition Examination Survey (NHANES) were analyzed using a complete-case approach, resulting in a final weighted sample of 192,331,773 adults. Multivariable linear and logistic regression models were applied to examine the relationship between antidepressant use and metabolic indicators, including BMI, fasting glucose, lipid profiles, and obesity. Analyses were adjusted for age, sex, race/ethnicity, and depression severity (Patient Health Questionnaire-9 {PHQ-9}), with subgroup analyses conducted by depression status and gender. All analyses accounted for NHANES's complex sampling design using Stata version 18 (College Station, TX: StataCorp LLC).

Results: Antidepressant use was not significantly associated with obesity in the full sample. However, in subgroup analyses, antidepressant use was linked to adverse metabolic changes, including higher total cholesterol and lower fasting blood glucose levels, particularly among non-depressed individuals and females. Significant racial/ethnic disparities were also observed.

Conclusion: While antidepressant use was not independently associated with obesity, it was linked to specific metabolic alterations, with effects varying by gender and depression status. These findings underscore the importance of monitoring metabolic health in antidepressant users, especially in a diverse population.

## Introduction

In recent times, obesity and depression are among the biggest health issues in the United States, and each inflicts significant cost and suffering on the people. The Centers for Disease Control and Prevention (CDC) data show that over 40% of US adults are obese, and between 7% and 10% experience a serious depressive episode each year [1]. Although the conditions are usually considered independently, research now points to a strong and complex connection between them [2]. Depression can make people less active, alter their eating habits, and disrupt their sleep, all of which tend to result in weight gain. In addition, obesity imposes excessive stress on humans and significantly increases their risk for developing depression [3,4]. The interrelationship makes caring for obese and depressed patients more difficult, especially when antidepressants or other drugs are used.

Treatment of moderate or severe depression usually starts with antidepressants, such as selective serotonin reuptake inhibitors (SSRIs), serotonin-norepinephrine reuptake inhibitors (SNRIs), and atypical antidepressants. They are becoming a cause for concern when it comes to how they may affect metabolic functions [5]. Some commonly prescribed antidepressants have been linked to side effects, such as weight gain, excessive and unhealthy eating habits, and glucose metabolism disorder [6]. These side effects compound the problem of obesity for antidepressant users and can complicate into other cardiometabolic issues, such as insulin resistance, dyslipidemia, and hypertension [7]. Although fluoxetine is sometimes associated with weight loss after initial use, people who take antidepressants for a long time usually gain considerable weight [8,9]. This makes the problem of obesity and depression difficult for patients and health professionals because psychological balance may put physical health at risk [10].

Although these connections are important in clinical practice, there is not a lot of population-level evidence about them. Many of the scientific studies are conducted using clinical trials or reviews of past studies that often do not have much diversity in age or income [11]. Also, few studies have considered in depth the many factors that impact both antidepressant use and weight gain, such as the severity of a person’s depression at onset, how active they are, their diet, other health constraints, and socioeconomic background [12,13]. The knowledge deficit is especially noticeable among polypharmacy patients as continuous antidepressant intake has become more common among the general population [14]. People with obesity and depression need to learn how certain drugs can affect their metabolism, since these effects can shape treatment decisions [15,16].

To deal with these gaps, large surveys, such as the National Health and Nutrition Examination Survey (NHANES), have become imperative. Since NHANES includes both self-reported behavior, physical examinations, and laboratory analyses, it is a great tool for examining the connections between mental health, medication use, and health burdens [17,18]. NHANES data were used in this study to examine the relationship between the use of antidepressants and obesity and its associated health factors in adults with depression in the United States [19]. This research aimed to ascertain if antidepressant usage directly affects weight-related outcomes or if these outcomes are mostly explained by problems related directly to depression and lifestyle [20]. Results from the research can provide useful information on managing depression and deciding on appropriate treatments. The study will further assess the association between antidepressant use and metabolic side effects, particularly obesity and related parameters, among US adults with depression using NHANES data [21].

## Materials and methods

Data source and study population

This cross-sectional study used data from the National Health and Nutrition Examination Survey (NHANES), covering seven continuous two-year cycles from 2005 to 2018 [21]. NHANES provides cross-sectional survey data by the National Center for Health Statistics (NCHS) using a complex, multistage probability sampling design to produce nationally representative estimates of US civilians and the non-institutionalized population.

We pooled data across 2005-2018 cycles to enhance statistical power through a large sample size and increase generalizability. Adults aged 18 years and older were included if they had complete data on key variables as follows: antidepressant use, depression symptoms, body mass index (BMI), waist circumference, and relevant metabolic biomarkers. Pregnant individuals were excluded.

Data handling and missing data strategy

Due to NHANES’s structured data collection approach, including laboratory subsampling protocols and questionnaire-specific skip patterns, some variables were not collected for all participants. Missing data were especially common in laboratory biomarkers, such as fasting glucose and lipid profiles, which were restricted to fasting subsamples, and in depression data when the Patient Health Questionnaire-9 (PHQ-9) was either not administered or incomplete.

To ensure internal consistency and avoid introducing bias through the imputation of non-randomly missing data, we performed a complete-case analysis. Only participants with non-missing data on all key exposure, outcome, and covariate variables were retained. This method is widely used in NHANES-based epidemiological research and was considered appropriate given that the missingness was structurally related to survey design rather than random data loss. As a result, our final analytic sample included 33,958 US adults, representing a weighted national population of 192,331,773 individuals.

Variables

Exposure Variable

The primary exposure was current antidepressant use, assessed through self-report of prescription medication use in the past 30 days, verified during the in-home interview and medication inventory review.

Outcome Variables

The primary outcome measures assessed in this study included both anthropometric and metabolic health indicators. Body mass index (BMI) in kg/m² was calculated using measured height and weight. Obesity was defined as a BMI equal to or greater than 30 kg/m², in accordance with CDC criteria. Additional outcome variables included waist circumference (cm), fasting glucose levels (mg/dL), high-density lipoprotein (HDL) cholesterol (mg/dL), triglycerides (mg/dL), and total cholesterol levels (mg/dL).

Covariates

All models were adjusted for several key covariates, including age (continuous, in years), sex (male/female), and race/ethnicity, categorized as non-Hispanic White, non-Hispanic Black, non-Hispanic Asian, and other Hispanic. Depression severity was measured using the Patient Health Questionnaire-9 (PHQ-9), with PHQ-9 scores incorporated both as continuous variables in the main analyses and as a binary indicator of depression (PHQ-9 ≥10) for subgroup analyses.

Statistical analysis

This study utilized Stata version 18 (College Station, TX: StataCorp LLC) for all statistical analyses. Descriptive statistics were generated to summarize the demographic and clinical characteristics of the study population, stratified by antidepressant use. To examine the relationship between antidepressant use and each outcome, we performed multivariable linear regression for continuous outcomes (e.g., BMI, lipid levels) and multivariable logistic regression for the binary outcome of obesity (BMI ≥30 kg/m²). All models were adjusted for relevant covariates and incorporated NHANES-provided sample weights, strata, and primary sampling units to account for the complex survey design, ensuring nationally representative estimates. Additionally, subgroup analyses were conducted by depression status and gender to assess potential effect modification.

Ethical considerations

The NHANES protocol was approved by the National Center for Health Statistics Research Ethics Review Board. All participants provided informed consent before data collection. Because this study used de-identified, publicly available data, it was deemed exempt from institutional review board (IRB) oversight.

## Results

This section presents the key findings of the study, organized into several subsections. First, the demographic and clinical characteristics of the study population are described, providing an overview of the sample composition. Next, the associations between antidepressant use and various metabolic outcomes, including body mass index, fasting glucose, lipid profiles, and obesity status, are reported using multivariable regression analyses. Following this, subgroup analyses explore whether these associations differ based on depression status and gender, highlighting potential variations in metabolic effects across these groups. All analyses account for the complex survey design and relevant covariates to ensure nationally representative and adjusted estimates.

Sample characteristics

Table 1 below shows the weighted distribution of sociodemographic and metabolic characteristics by antidepressant use among US adults. Table 1 shows that the average age of antidepressant users was slightly lower (53.79±12.54) years than that of non-users (55.55±16.16) years, with this difference statistically significant (p<0.001). Notably, the mean PHQ-9 score, a validated measure of depression severity, was considerably higher among antidepressant users (5.85±4.94) compared to non-users (3.69±4.64), and the difference was significant at 5% (p<0.001), confirming a greater burden of depressive symptoms in the group.

**Table 1:** Weighted distribution of sociodemographic and metabolic characteristics by antidepressant use among US adults (NHANES 2005-2018; unweighted n=33,958; weighted n=192,331,773). *P<0.001 is statistically significant. **P<0.05 is statistically significant. Values are presented as weighted means±standard deviation for continuous variables and weighted frequencies (percentages) for categorical variables. Statistical comparisons were conducted using design-based F-tests for categorical variables and t-tests for continuous variables, accounting for the complex survey design and sample weights of NHANES. NHANES: National Health and Nutrition Examination Survey; HDL: high-density lipoprotein; PHQ-9: Patient Health Questionnaire-9

Variables	Category	Non-antidepressant use	Antidepressant use	t-test	Chi-square	p-Value
Age (in years)	-	55.55±16.16	53.79±12.54	3.74	-	p<0.001*
PHQ-9 score	-	3.69±4.64	5.85±4.94	-10.44	-	p<0.001*
Body mass index (kg/m^2^)	-	30.12±7.27	30.46±6.36	-1.44	-	0.152
Waist circumference (cm)	-	103.17±17.43	102.95±14.65	0.43	-	0.666
Fasting glucose (mg/dL)	-	113.96±39.42	108.19±29.70	5.64	-	p<0.001*
Direct HDL-cholesterol (mg/dL)	-	53.69±17.17	55.08±14.98	-2.38	-	0.019**
Triglyceride (mg/dL)	-	139.25±113.82	148.70±104.85	-2.32	-	0.022**
Total cholesterol (mg/dL)	-	189.64±42.96	200.68±40.46	-7.01	-	p<0.001*
Obese	Non-obese	101,327,601 (95%)	4,921,546 (5%)	-	4.55	0.122
	Obese	80,619,075 (95%)	4,315,790 (5%)	-		
Gender	Male	101,327,601 (96%)	4,444,288 (4%)	-	32.79	p<0.001*
	Female	81,766,836 (94%)	4,793,048 (6%)	-		
Race/ethnicity	Other Hispanic	10,575,693 (98%)	220,213 (2%)	-	126.12	p<0.001*
	Non-Hispanic White	7,637,268 (97%)	234,037 (3%)	-		
	Non-Hispanic Black	140,000,000 (94%)	7,971,610 (6%)	-		
	Non-Hispanic Asian	18,524,459 (98%)	440,576 (2%)	-		
	Other race	10,578,027 (97%)	370,900 (3%)	-		

Concerning metabolic parameters, no statistically significant difference was observed in body mass index (BMI) or waist circumference between the groups, indicating similar overall adiposity profiles (BMI: 30.46±6.36 vs. 30.12±7.27 kg/m², p=0.15; waist circumference: 102.95±14.65 vs. 103.17±17.43 cm, p=0.67). However, several cardiometabolic biomarkers differed significantly. Antidepressant users had lower mean fasting glucose levels (108.19±29.70 vs. 113.96±39.42 mg/dL, p<0.001), higher HDL-cholesterol (55.08±14.98 vs. 53.69±17.17 mg/dL, p=0.02), and higher triglyceride levels (148.70±104.85 vs. 139.25±113.82 mg/dL, p=0.02). Additionally, total cholesterol was significantly elevated in the antidepressant group (200.68±40.46 vs. 189.64±42.96 mg/dL, p<0.001), suggesting a mixed metabolic profile associated with antidepressant use. Table 2 below indicates the key findings comparing the metabolic parameters between antidepressant users and non-users.

**Table 2:** Comparison of metabolic parameters between antidepressant users and non-users. BMI: body mass index; ↑: significantly higher; ↓: significantly lower; HDL-C: high-density lipoprotein cholesterol; NS: not significant

Parameters	Antidepressant users	Non-users	p-Value	Significance
BMI (kg/m²)	30.46±6.36	30.12±7.27	0.15	NS
Waist circumference (cm)	102.95±14.65	103.17±17.43	0.67	NS
Fasting glucose (mg/dL)	108.19±29.70	113.96±39.42	<0.001	↓ in users
HDL-C (mg/dL)	55.08±14.98	53.69±17.17	0.02	↑ in users
Triglycerides (mg/dL)	148.70±104.85	139.25±113.82	0.02	↑ in users
Total cholesterol (mg/dL)	200.68±40.46	189.64±42.96	<0.001	↑ in users

Although the prevalence of obesity did not differ significantly by antidepressant use (χ²=4.55, p=0.12), gender and racial/ethnic distributions did. A higher proportion of antidepressant users were female, 4,793,048 (6%) vs. 4,444,288 (4%) among males, statistically significant at p<0.001. Racial/ethnic variation in antidepressant use was also significant (p<0.001), with non-Hispanic Black adults showing the highest prevalence, 7,971,610 (6%) among users, followed by non-Hispanic Asians, 440,576 (2%), and other groups with lower percentages. These disparities highlight the importance of considering both social and biological determinants in understanding antidepressant-related metabolic outcomes.

These key findings have disclosed distinct antidepressant use metabolic effects regardless of the comparable adiposity measures noted between the groups. Even though antidepressant users had lower fasting glucose levels and higher HDL-cholesterol, possibly indicating improvements in glycemic control alongside favorable lipid modulation, the elevation of triglycerides and total cholesterol is indicative of conflicting cardiometabolic effects. Such a mixed profile implies that the antidepressants might differentially affect the metabolic pathways, potentially as a result of medication-specific mechanisms. Clinically, these findings indicate the requirement for individualized evaluation and monitoring of glucose and lipid parameters in patients using antidepressants, given that their net cardiovascular risk is likely to differ despite the neutral impact on body weight. Additional studies on drug-specific metabolic outcomes are needed to optimize the selection of treatment, especially in individuals with cardiovascular diseases or pre-existing metabolic syndrome.

Association between antidepressant use and metabolic outcomes (multivariable regression analysis)

Table 3 below shows findings on the association between antidepressant use and metabolic parameters among US adults with depression. Table 3 shows that antidepressant use was significantly associated with lower fasting glucose levels (β=-5.098, p<0.001), even after accounting for potential confounders. This finding may reflect better glycemic monitoring or healthcare engagement among users, although causal interpretation remains limited.

**Table 3:** Association between antidepressant use and metabolic parameters among US adults with depression: multivariable linear regression models (NHANES 2005-2018; unweighted n=33,958; weighted n=192,331,773). *P<0.001 is statistically significant. **P<0.05 is statistically significant. ***P<0.01 is statistically significant. This table presents adjusted beta coefficients and standard errors (in parentheses) from multivariable linear regression models assessing the association between antidepressant use and various metabolic parameters, including BMI, fasting glucose, HDL cholesterol, triglycerides, total cholesterol, and waist circumference. All models accounted for survey design and sample weights to ensure nationally representative estimates. Covariates included PHQ-9 score, age, gender, and race/ethnicity. The table is compiled by the authors from the NHANES survey data. SE: standard error; NHANES: National Health and Nutrition Examination Survey; HDL: high-density lipoprotein; PHQ-9: Patient Health Questionnaire-9

Variables	Body mass index (SE) (kg/m^2^)	Fasting glucose (SE) (mg/dL)	Direct HDL-cholesterol (SE) (mg/dL)	Triglyceride (SE) (mg/dL)	Total cholesterol (SE) (mg/dL)	Waist circumference (SE) (cm)
Antidepressant use	0.125 (0.242)	-5.098^* ^(0.948)	1.574^*** ^(0.543)	4.259 (3.821)	9.349^* ^(1.517)	-0.634 (0.497)
PHQ-9 score	0.142^* ^(0.023)	0.382^*** ^(0.139)	-0.124 (0.093)	1.858^* ^(0.346)	0.289 (0.163)	0.309^* ^(0.056)
Age (in years)	0.003 (0.007)	0.380^* ^(0.033)	0.079^*^(0.018)	0.072 (0.102)	-0.099^** ^(0.038)	0.127^* ^(0.017)
Female	0.343 (0.292)	-0.884 (1.387)	2.855^* ^(0.673)	-3.074 (3.392)	6.069^* ^(1.518)	-0.900 (0.696)
Race/ethnicity (vs. other Hispanic)	-0.811^** ^(0.389)	0.223 (2.966)	-0.508 (0.655)	4.180 (10.367)	0.696 (2.373)	-2.438^*** ^(0.875)
Race/ethnicity (vs. non-Hispanic White)	-0.537 (0.272)	-8.099^* ^(1.450)	2.202^*^(0.576)	-5.082 (4.321)	0.026 (1.466)	0.579 (0.632)
Race/ethnicity (vs. non-Hispanic Black)	1.693^* ^(0.324)	-2.273 (1.924)	4.473^* ^(0.676)	-39.988^* ^(4.541)	-6.418^* ^(1.720)	2.773^* ^(0.787)
Race/ethnicity (vs. non-Hispanic Asian)	-2.083^* ^(0.539)	-5.446^** ^(2.465)	1.056 (0.958)	-3.813 (7.191)	-6.178^** ^(2.631)	-4.000^*** ^(1.399)
Constant	29.680^* ^(0.446)	98.406^* ^(1.868)	46.348^* ^(0.848)	137.633^* ^(5.010)	192.336^* ^(2.578)	94.983^* ^(1.030)
Observations	33958	33958	33958	33958	33958	33958
R^2^	0.022	0.029	0.018	0.015	0.013	0.028

Users of antidepressants also had significantly higher HDL-cholesterol levels (β=1.57, p=0.004) and higher total cholesterol (β=9.35, p<0.001), suggesting a complex lipid profile that includes both protective and adverse elements. Although triglycerides were higher among antidepressant users (β=4.26), this association was not statistically significant. Similarly, antidepressant use was not significantly associated with waist circumference or BMI, suggesting that, after adjustment, these general adiposity measures do not differ meaningfully between users and non-users in this sample.

The severity of depression (PHQ-9 score) was positively associated with several metabolic indicators. Higher depression scores predicted greater BMI (β=0.14, p<0.001), fasting glucose (β=0.38, p<0.01), triglycerides (β=1.86, p<0.001), and waist circumference (β=0.31, p<0.001), highlighting the potential metabolic burden of depressive symptoms themselves.

Demographic differences were also apparent. Non-Hispanic Black individuals showed significantly higher BMI and HDL but markedly lower triglycerides and total cholesterol compared to other Hispanics. Non-Hispanic Asians had significantly lower BMI, glucose, and total cholesterol than the reference group. These racial/ethnic disparities emphasize the importance of stratified analyses and culturally informed interventions in addressing metabolic health.

Despite the relatively low R² values across models (ranging from 0.013 to 0.029), which is typical for population-level cross-sectional health data, the findings reveal important independent associations between antidepressant use and select metabolic outcomes, suggesting that treatment for depression may carry both risks and potential benefits in the context of metabolic health.

Association between antidepressant use and obesity (logistic regression analysis)

Table 4 below indicates the findings regarding the association between antidepressant use and obesity among US adults with depression. In this multivariable logistic regression model, the association between antidepressant use and obesity was assessed while controlling for depression severity, age, gender, and race/ethnicity (Table 4). The results indicate that antidepressant use was not significantly associated with obesity (OR=1.07; 95% CI: 0.92-1.23; p=0.38), suggesting that after adjusting for relevant covariates, antidepressant treatment does not independently increase the odds of being classified as obese in this nationally representative sample.

**Table 4:** Association between antidepressant use and obesity among US adults with depression: multivariable logistic regression model (NHANES 2005-2018; unweighted n=33,958; weighted n=192,331,773). *P<0.001 is statistically significant. **P<0.05 is statistically significant. ***P<0.01 is statistically significant. This table presents odds ratios (ORs), 95% confidence intervals (CIs), and p-values from a multivariable logistic regression model evaluating the association between antidepressant use and obesity among adults with depression. The analysis incorporated NHANES survey weights and design variables to produce nationally representative estimates. Covariates included PHQ-9 score, age, sex, and race/ethnicity. The table is compiled by the authors from the NHANES survey data. NHANES: National Health and Nutrition Examination Survey; PHQ-9: Patient Health Questionnaire-9

Obese	OR	95% CI	p-Value
Antidepressant use	1.07	0.92-1.23	0.384
PHQ-9 score	1.04	1.02-1.05	<0.001*
Age (in years)	1.00	1.00-1.01	0.462
Female	1.03	0.89-1.20	0.647
Race/ethnicity vs. other Hispanic	0.75	0.60-0.93	0.010**
Race/ethnicity vs. non-Hispanic White	0.80	0.67-0.94	0.009***
Race/ethnicity vs. non-Hispanic Black	1.30	1.10-1.54	0.002***
Race/ethnicity vs. non-Hispanic Asian	0.61	0.44-0.85	0.004***

In contrast, depression severity (PHQ-9 score) showed a significant positive association with obesity (OR=1.04; 95% CI: 1.02-1.05; p<0.001), implying that individuals with more severe depressive symptoms are more likely to be obese, regardless of antidepressant use. This finding supports the growing body of evidence linking psychological distress with adverse metabolic outcomes.

Gender and age were not significantly associated with obesity in this adjusted model. However, racial/ethnic disparities were pronounced. Compared to other Hispanics, non-Hispanic Black participants had 30% higher odds of obesity (OR=1.30; 95% CI: 1.10-1.54; p=0.002), while non-Hispanic Asians had significantly lower odds (OR=0.61; 95% CI: 0.44-0.85; p=0.004). Similarly, non-Hispanic Whites and individuals of "other race" categories also had lower odds of obesity compared to other Hispanics. These disparities highlight the importance of accounting for racial/ethnic background in understanding the distribution of obesity in the context of mental health and medication use.

Analysis of antidepressant use and metabolic outcomes stratified by depression status (depressed vs. non-depressed)

Table 5 below shows the findings of subgroup analyses with regard to the association between antidepressant use and metabolic markers stratified by depression status. The stratified analysis examined whether the relationship between antidepressant use and metabolic health indicators varied by depression status. Among individuals with depression, antidepressant use was associated with significantly lower fasting glucose levels (β=-4.49, p<0.01) and modestly higher total cholesterol (β=8.12, p<0.05). However, no significant association was observed between antidepressant use and BMI, HDL-cholesterol, triglycerides, or waist circumference in this group. This suggests a potentially favorable effect of antidepressant use on glycemic regulation in depressed individuals, though some compensatory elevation in total cholesterol may occur.

**Table 5:** Subgroup analyses: association between antidepressant use and metabolic markers stratified by depression status (NHANES 2005-2018; weighted population n=192,331,773). *P<0.001 is statistically significant. **P<0.01 is statistically significant. ***P<0.05 is statistically significant. This table presents beta coefficients and standard errors (in parentheses) from multivariable linear regression models stratified by depression status (depressed vs. non-depressed), examining the association between antidepressant use and six metabolic outcomes as follows: body mass index (BMI), fasting glucose, HDL cholesterol, triglycerides, total cholesterol, and waist circumference. Models were adjusted for age, gender, and race/ethnicity and accounted for NHANES' complex survey design using sampling weights. The analysis was conducted separately for individuals identified as depressed (n=7,871) and non-depressed (n=8,728) based on PHQ-9 scores. This table is compiled by the authors from NHANES survey data. NHANES: National Health and Nutrition Examination Survey; HDL: high-density lipoprotein; PHQ-9: Patient Health Questionnaire-9; SE: standard error

Depressed (n=7871)							Non-depressed (n=8728)					
Variables	Body mass index (SE) (kg/m^2^)	Fasting glucose (SE) (mg/dL)	Direct HDL-cholesterol (SE) (mg/dL)	Triglyceride (SE) (mg/dL)	Total cholesterol (SE) (mg/dL)	Waist circumference (SE) (cm)	Body mass index (SE) (kg/m^2^)	Fasting glucose (SE) (mg/dL)	Direct HDL-cholesterol (SE) (mg/dL)	Triglyceride (SE) (mg/dL)	Total cholesterol (SE) (mg/dL)	Waist circumference (SE) (cm)
Antidepressant use	0.163 (0.418)	-4.489^** ^(1.651)	-0.344 (0.879)	9.340 (9.209)	8.123^*** ^(3.517)	-0.246 (0.946)	0.271 (0.278)	-5.075^* ^(1.140)	1.945^** ^(0.643)	5.280 (3.861)	10.063^* ^(1.621)	-0.456 (0.574)
Age (in years)	-0.014 (0.024)	0.070 (0.213)	0.151^* ^(0.036)	-0.149 (0.242)	-0.028 (0.138)	0.073 (0.055)	0.004 (0.007)	0.408^* ^(0.029)	0.073^* ^(0.019)	0.083 (0.107)	-0.109^** ^(0.035)	0.131^* ^(0.018)
Female	1.280 (0.919)	1.511 (4.440)	0.632 (3.016)	-10.464 (11.830)	-1.175 (5.098)	1.031 (2.325)	0.278 (0.297)	-1.080 (1.452)	3.084^* ^(0.640)	-1.240 (3.858)	7.113^* ^(1.553)	-1.043 (0.709)
Race/ethnicity (vs. other Hispanic)	-1.023 (1.180)	-8.692 (8.649)	-2.628 (1.744)	-3.945 (24.221)	-1.933 (6.137)	-2.269 (2.463)	-0.722 (0.427)	0.866 (2.589)	-0.111 (0.755)	5.613 (11.367)	0.947 (2.599)	-2.335^***^ (0.971)
Race/ethnicity (vs. non-Hispanic White)	-0.422 (1.067)	-23.174^* ^(6.817)	2.765 (1.844)	-19.185 (22.239)	-6.834 (5.490)	1.620 (2.200)	-0.537 (0.297)	-6.255^* ^(1.434)	2.116^* ^(0.593)	-3.236 (3.884)	0.892 (1.444)	0.476 (0.684)
Race/ethnicity (vs. non-Hispanic Black)	1.069 (1.266)	-19.579^** ^(7.293)	3.895^*** ^(1.796)	-53.519^*** ^(22.200)	-15.723^** ^(5.963)	2.461 (2.675)	1.816^* ^(0.341)	-0.062 (1.829)	4.567^* ^(0.723)	-37.910^* ^(4.034)	-5.025^** ^(1.688)	2.888^* ^(0.812)
Race/ethnicity (vs. non-Hispanic Asian)	-1.604 (1.614)	-27.744^* ^(7.292)	1.067 (2.633)	-17.556 (31.783)	-2.091 (9.700)	-0.925 (3.904)	-2.164^* ^(0.599)	-2.878 (2.683)	1.083 (1.075)	-2.489 (8.358)	-6.481^*** ^(2.519)	-4.394^** ^(1.547)
Constant	31.945^* ^(1.626)	134.271^* ^(11.928)	42.160^* ^(2.718)	188.980^* ^(23.930)	202.127^* ^(9.609)	100.361^* ^(3.661)	29.999^* ^(0.448)	95.970^* ^(1.760)	46.277^* ^(0.947)	139.435^* ^(5.403)	192.415^* ^(2.221)	95.647^* ^(1.062)

In contrast, among those without depression, antidepressant use was similarly associated with significantly lower fasting glucose levels (β=-5.08, p<0.001), higher HDL-cholesterol (β=1.95, p<0.01), and higher total cholesterol (β=10.06, p<0.001). Again, no significant relationship was observed for BMI, triglycerides, or waist circumference. These findings indicate that antidepressant use is consistently associated with more favorable glucose profiles, regardless of depression status, and may also improve HDL levels in non-depressed individuals. However, the increase in total cholesterol across both groups raises questions about potential lipid-related side effects that warrant further monitoring.

Moreover, the subgroup results highlight important differences in racial/ethnic metabolic profiles. Notably, non-Hispanic Black adults had significantly higher HDL and significantly lower triglyceride levels compared to most other racial/ethnic groups, consistent with known epidemiologic patterns. Interestingly, non-Hispanic Asians consistently exhibited lower BMI and waist circumference, further supporting existing literature on body composition differences across ethnicities.

Analysis of the association between antidepressant use and metabolic outcomes stratified by sex (male vs. female)

Table 6 shows the findings of the subgroup analyses regarding the association between antidepressant use and metabolic outcomes stratified by gender. Table 6 shows that gender-stratified analyses reveal important differences in the metabolic effects of antidepressant use. Among males, antidepressant use was significantly associated with lower fasting glucose (β=-3.796, p<0.01), higher HDL-cholesterol (β=1.930, p<0.05), and elevated total cholesterol (β=11.568, p<0.001). However, antidepressant use was not significantly related to BMI, triglycerides, or waist circumference in this group. These findings suggest that for men, antidepressants may offer favorable effects on glycemic and HDL profiles, but may contribute to elevated total cholesterol, highlighting a potential trade-off.

**Table 6:** Subgroup analyses: association between antidepressant use and metabolic outcomes stratified by gender (NHANES 2005-2018; weighted population n=192,331,773). *P<0.01 is statistically significant. **P<0.05 is statistically significant. ***P<0.001 is statistically significant. This table presents beta coefficients and standard errors (in parentheses) from multivariable linear regression models assessing the association between antidepressant use and six metabolic outcomes (body mass index, fasting glucose, HDL cholesterol, triglycerides, total cholesterol, and waist circumference), stratified by gender (male and female). Each model was adjusted for age, race/ethnicity, and depression status (PHQ-9), and accounted for the NHANES complex survey design and sampling weights to ensure national representativeness. This table is compiled by the authors from NHANES survey data. NHANES: National Health and Nutrition Examination Survey; HDL: high-density lipoprotein; PHQ-9: Patient Health Questionnaire-9; SE: standard error

Male							Female					
Variables	Body mass index (SE) (kg/m^2^)	Fasting glucose (SE) (mg/dL)	Direct HDL-cholesterol (SE) (mg/dL)	Triglyceride (SE) (mg/dL)	Total cholesterol (SE) (mg/dL)	Waist circumference (SE) (cm)	Body mass Index (SE) (kg/m^2^)	Fasting glucose (SE) (mg/dL)	Direct HDL-cholesterol (SE) (mg/dL)	Triglyceride (SE) (mg/dL)	Total cholesterol (SE) (mg/dL)	Waist circumference (SE) (cm)
Antidepressant use	0.289 (0.335)	-3.796^*^ (1.385)	1.930^**^ (0.892)	5.648 (4.648)	11.568^***^ (2.268)	-0.651 (0.788)	0.242 (0.344)	-5.813^***^ (1.347)	0.988 (0.746)	6.612 (5.029)	7.794^***^ (2.198)	-0.102 (0.788)
Depression	1.016 (0.715)	4.021 (2.953)	0.154 (2.779)	22.922^**^ (9.552)	6.798 (3.831)	2.612 (1.646)	1.955^***^ (0.520)	6.238 (3.833)	-2.347^**^ (1.039)	13.573 (7.592)	-0.835 (3.740)	4.509^***^ (1.326)
Age (in years)	0.003 (0.011)	0.433^***^ (0.043)	0.060^**^ (0.028)	0.121 (0.146)	-0.250^***^ (0.051)	0.150^***^ (0.027)	0.001 (0.009)	0.314^***^ (0.060)	0.104^***^ (0.018)	-0.005 (0.133)	0.076 (0.051)	0.098^***^ (0.022)
Race/ethnicity (vs. other Hispanic)	-0.790 (0.461)	-2.259 (3.924)	-0.594 (1.042)	1.156 (17.026)	-0.166 (3.280)	-2.112 (1.076)	-0.729 (0.599)	3.282 (4.023)	-0.437 (0.911)	9.552 (9.901)	2.538 (2.831)	-2.687^**^ (1.274)
Race/ethnicity (vs. non-Hispanic White)	-0.570 (0.386)	-9.487^***^ (2.116)	2.132^*^ (0.740)	-10.056 (6.093)	-1.237 (1.895)	0.334 (0.908)	-0.461 (0.519)	-6.452^**^ (2.897)	2.333^**^ (0.953)	1.351 (5.794)	2.348 (2.298)	0.858 (1.146)
Race/ethnicity (vs. non-Hispanic Black)	1.079^*^ (0.402)	-3.597 (2.561)	3.002^*^ (0.917)	-38.696^***^ (6.567)	-7.985^***^ (2.105)	1.632 (0.889)	2.242^***^ (0.564)	-0.884 (2.875)	5.757^***^ (0.951)	-38.696^***^ (5.498)	-3.727 (2.589)	3.741^*^ (1.210)
Race/ethnicity (vs. non-Hispanic Asian)	-2.071^**^ (0.821)	-3.827 (3.050)	1.041 (1.374)	-0.103 (9.566)	-3.883 (3.102)	-4.211 (2.229)	-2.170^*^ (0.715)	-7.829^**^ (3.624)	1.298 (1.257)	-9.337 (10.259)	-8.076 (4.397)	-4.016^**^ (1.728)
Constant	30.110^***^ (0.661)	97.474^***^ (2.255)	47.141^***^ (1.318)	142.603^***^ (7.410)	201.975^***^ (3.058)	94.778^***^ (1.505)	30.325^***^ (0.476)	100.567^***^ (3.399)	47.409^***^ (1.096)	139.981^***^ (8.529)	188.231^***^ (3.226)	96.051^***^ (1.144)

Among females, antidepressant use was also significantly associated with lower fasting glucose levels (β=-5.81, p<0.001) and higher total cholesterol (β=7.79, SE=2.198, p<0.001). However, unlike in males, the association with HDL-cholesterol was not statistically significant. No significant effects were observed for BMI, triglycerides, or waist circumference. This suggests that the metabolic profile of antidepressant use in women is primarily characterized by improved glucose control and a modest increase in total cholesterol, but with less pronounced HDL benefits compared to men.

Across both genders, the use of antidepressants does not appear to be linked to weight gain or increased adiposity, as reflected by non-significant associations with BMI and waist circumference. These results challenge common assumptions about widespread weight-related side effects from antidepressants and suggest that metabolic effects may be more nuanced and sex-specific, with glucose regulation improving in both groups, but lipid changes varying by gender.

Notably, race/ethnicity continued to demonstrate strong independent effects on metabolic markers. Non-Hispanic Black and non-Hispanic Asian participants of both genders had significantly different lipid profiles and body composition compared to reference groups, reaffirming the need to account for these covariates in clinical assessments and future research.

## Discussion

This study investigated the association between antidepressant use and metabolic outcomes among US adults using NHANES data from 2005 to 2018. After controlling for clinical and demographic factors, we did not find a significant correlation between antidepressant use and obesity. However, the severity of depression, as measured by the PHQ-9 score, was linked to a higher chance of obesity, suggesting that depression itself, rather than the medications used to treat it, might have a bigger impact on unhealthy metabolic conditions.

Subgroup analyses revealed notable differences by depression status and gender. Among non-depressed individuals, antidepressant use was significantly associated with increased total cholesterol and decreased fasting glucose levels, while in depressed individuals, associations with metabolic markers were less consistent. Gender-specific results also showed that women using antidepressants had significantly lower fasting glucose levels and higher total cholesterol, whereas men exhibited higher HDL-cholesterol and total cholesterol but reduced fasting glucose. These differences suggest that the metabolic effects of antidepressants may vary based on both depression status and biological sex, which points to the importance of tailored clinical monitoring.

Racial and ethnic disparities were also evident across models, with non-Hispanic Black and Asian participants consistently demonstrating distinct metabolic patterns compared to non-Hispanic Whites and Hispanics. These differences persisted after controlling for antidepressant use and depression severity, which emphasizes the value of incorporating social, environmental, and genetic factors into models of metabolic health and treatment response. Overall, while antidepressants did not independently increase obesity risk, their association with select metabolic parameters warrants attention, particularly in populations at higher risk for cardiovascular or metabolic disease. Our findings highlight the importance of looking closely at how obesity and depression are connected through metabolic and biological processes on their own, instead of just focusing on drug treatments. The analysis of the bidirectional relationship between neurotransmitters responsible for depressive symptoms and key metabolic hormonal regulators on causal factors, such as impaired satiety, food seeking behaviors, and cravings, sleep patterns, and fat tissue metabolism, may explain the differences across various subgroups of individuals. These should be further explored in future longitudinal studies to clarify causality and explore underlying mechanisms to proffer solutions to managing this rising global pandemic.

Additionally, there seems to be conflicting evidence about the effect of age, gender, and ethnic groups on the relationship between depression, obesity, and antidepressants. Few studies propose that depression developed during adolescence is significantly related to obesity in adulthood, while some others have shown that depression diagnosed in adults has no association with obesity in adulthood [22]. The current study has shown that age is a significant predictor of some metabolic outcomes; however, there is no data about the age of onset of depression for our sample population. Furthermore, our study group showed that about 6% of the female population use antidepressants, while only 4% of the male population use antidepressants. This is in keeping with the hypothesis that women are more likely to be depressed than men [23]. Li et al. found that depression was directly related to various measures of obesity in women, but not in men [23]. The current study shows a significant effect of race on the relationship between antidepressant use, depression, and obesity, with a higher prevalence in non-Hispanic blacks. In contrast, a study by Polanka et al. found a greater relationship in Hispanics/Latinos than non-Hispanic whites and blacks [24]. This may probably be due to the non-Hispanic black population of our sample being more users of antidepressants (6%).

Study limitations

Despite the strengths of using a large, nationally representative dataset and robust modeling strategies, this study has several important limitations. A key limitation is the presence of missing data across multiple metabolic and demographic variables, which were not missing at random (NMAR). Due to this non-random pattern of omission, we employed complete case analysis, which may have introduced selection bias and reduced the generalizability of our results. While this approach ensured internal consistency and reduced imputation-related errors, it also potentially excluded individuals with more complex health profiles or lower socioeconomic status, who are both more likely to have missing data and to experience adverse metabolic outcomes. Additionally, the cross-sectional nature of NHANES data precludes causal inference, limiting our ability to determine whether antidepressant use directly influences metabolic outcomes or whether shared underlying factors, such as health behaviors or comorbidities, may account for observed associations.

Another limitation is the inability to distinguish between classes of antidepressants, which vary in their metabolic side-effect profiles. For instance, selective serotonin reuptake inhibitors (SSRIs) may have different cardiometabolic effects compared to atypical antidepressants or tricyclics. Furthermore, dosage, treatment duration, and adherence were not captured in the available data, limiting our ability to conduct dose-response analyses. Finally, residual confounding is possible despite multivariable adjustment, particularly for behavioral and dietary factors that were not fully accounted for in our models.

To tackle these limitations, prospective longitudinal studies need to prioritize numerous key strategies. Firstly, prospective research designs with repeated metabolic evaluations and measurements may aid in clarifying the temporal relationships between the use of antidepressants and cardiometabolic changes, in addition to assisting in differentiating between confounding and causation. The integration of sensitivity analyses and multiple imputation for missing data was also aimed at enhancing generalizability by accounting for non-random omissions, especially in high-risk subgroups. Still, prospective longitudinal studies should strive to stratify the findings based on the antidepressant class and use duration, given that metabolic effects might significantly vary based on exposure duration and drug type. Lastly, to tackle the residual confounding, longitudinal studies should integrate biomarker or genetic-based metabolic risk proxies and granular behavioral data, including sleep, physical activity, and diet data. Lastly, leveraging the causal inference methods that include Mendelian randomization and propensity score matching might reinforce evidence for direct effects of medication. Such advancements are likely to inform individualized prescriptions for persons with depression and comorbid metabolic conditions.

## Conclusions

This study found that antidepressant use was not significantly associated with obesity but was linked to key metabolic changes, including lower fasting glucose and higher total cholesterol levels. These associations varied by gender and depression status, with stronger cholesterol effects observed in women and non-depressed individuals. Additionally, depressive symptoms were independently associated with a higher risk of obesity. These findings highlight the need for routine metabolic monitoring in patients using antidepressants and underscore the complex relationship between mental health treatment and physical health outcomes.

Findings herein show a statistically significant relationship between depression and obesity, which was corroborated by previous literature. The authors hinted that the proper management of depressive symptoms can lead to a decline in obesity, and conversely, that addressing obesity may help alleviate depressive symptoms. Depression and obesity provoke dysfunction of the endocrine system and the hypothalamic-pituitary-adrenal pathway, invoking inflammation and its mediators. While our findings did not show a direct association between the use of antidepressants alone and obesity, some have indicated otherwise. It is imperative to note that antidepressants illicit varying responses in patients based on the different drug classes. For instance, most members of serotonin and norepinephrine reuptake inhibitors show minimal effect on BMI except after prolonged use, tricyclic antidepressants have a greater tendency for rapid increased weight gain. Furthermore, most individuals with depression and comorbid obesity tend to be patients with existing medical conditions, which are managed with separate medications, further complicating the situation with polypharmacy.
